# Design and Study of Novel Composites Based on EPDM Rubber Containing Bismuth (III) Oxide and Graphene Nanoplatelets for Gamma Radiation Shielding

**DOI:** 10.3390/polym16050633

**Published:** 2024-02-26

**Authors:** Gabriela Álvarez-Cortez, Francisco Molina, Bruno F. Urbano, Mohamed Dahrouch, Marianella Hernández Santana, Miguel A. Lopez Manchado, Raquel Verdejo, Héctor Aguilar Bolados

**Affiliations:** 1 Departamento de Polímeros, Facultad de Ciencias Químicas, Universidad de Concepción, Concepcion 3349001, Chile; galvarez@udec.cl (G.Á.-C.); burbano@udec.cl (B.F.U.); 2 Centro de Investigación en Física Nuclear y Espectroscopia de Neutrones CEFNen, Comisión Chilena de Energía Nuclear, Santiago 7600713, Chile; francisco.molina@cchen.cl; 3 Millennium Institute for Subatomic Physics at High Energy Frontier—SAPHIR, Santiago 7591538, Chile; 4 Departamento de Química Orgánica, Facultad de Ciencias Químicas, Universidad de Concepción, Concepcion 3349001, Chile; 5 Instituto de Ciencia y Tecnología de Polímeros (ICTP), CSIC, Juan de la Cierva, 3, 28006 Madrid, Spainlmanchado@ictp.csic.es (M.A.L.M.);

**Keywords:** EPDM rubber, bismuth (III) oxide, graphene nanoplatelets, gamma radiation attenuation coefficient

## Abstract

The mechanical, thermal and gamma radiation attenuation properties of ethylene–propylene–diene monomer (EPDM)-based composites containing graphene nanoplatelets (GNs) and bismuth (III) oxide nanoparticles (B) were investigated. The use of polyethylene glycol (PEG) as a compatibilizer to improve the dispersion of the fillers was also investigated. The results showed that the combined use of these fillers resulted in a drastic increase in mechanical properties, reaching 123% and 83% of tensile strength and elongation at break, respectively, compared to those of EPDM. In contrast, the addition of PEG to composites containing EPDM GNs and B resulted in composites with lower values of mechanical properties compared to the EPDM/B/GN-based composite. However, the presence of PEG leads to obtaining a composite (EPDM/B/GNP) with a mass attenuation coefficient to gamma radiation (^137^Cs, 662 keV) superior to that composite without PEG. In addition, the composite EPDM, B and PEG exhibited an elongation at break 153% superior to unfilled EPDM. Moreover, the binary filler system consisting of 100 phr of bismuth (III) oxide and 10 phr of GN leads to reaching 61% of the linear damping coefficient of the EPDM composite compared to that value of the unfilled EPDM. The study of the morphology and the state of filler dispersion in the polymer matrix, obtained using scanning electron microscopy and energy-dispersive X-ray spectroscopy, respectively, provides a useful background for understanding the factors affecting the gamma radiation attenuation properties. Finally, the results also indicated that by adjusting the formulation, it is possible to tune the mechanical and thermal properties of EPDM composites reinforced with bismuth oxide and graphene nanoplatelets.

## 1. Introduction

The rubber industry has explored a wide range of additives and modifiers to optimize the behavior of these materials for their various applications, improving their respective properties and performance. For example, one of the most interesting applications described in the literature is the use of fillers to impart better mechanical properties, such as carbon black, silica, organoclays and graphene materials, among others [1,2,3,4].

Nowadays, one of the current challenges in the high-tech industry is related to high-energy radiation and its interaction with matter in areas such as medical radiology, radiation protection and observational astronomy. The use of rubbers in the production of materials that allow attenuation of this radiation, combined with flexibility properties, can be oriented to versatile applications such as flexible shielding and coating of electronic devices, among others [5]. As it is well known [6], lead is the metallic element most often used for radiation attenuation, but this element is toxic both for human health and for the environment. In this context, the bismuth element seems to emerge as a viable alternative to replace lead since it is more environmentally friendly and non-toxic to health [7,8,9]. Studies show that bismuth oxide (Bi_2_O_3_) has adequate properties to attenuate the high-energy radiation. It has been reported [10] that in a natural rubber matrix, the addition of fillers composed of metal oxides containing a metal element with a high atomic number, such as magnetite (Fe_3_O_4_, Z(Fe) = 26), tungsten oxide (W_2_O_3_, Z(W) = 74) and bismuth oxide (Bi_2_O_3_, Z(Bi) = 83), showed the ability to attenuate gamma radiation. Based on this study, the best performance was obtained using Bi_2_O_3_ filler. Similarly, Thumwong A. et al. [11] showed that the addition of bismuth (III) oxide nanoparticles to natural rubber in different proportions (50 to 100, 150 and 200 phr) resulted in greater attenuation of X-rays, and, in addition, an increase in mechanical properties was observed. Another similar study described that the combination of ethylene–propylene–diene monomer (EPDM) rubber as a matrix and bismuth (III) oxide nanomaterial formed a material capable of reducing transmitted gamma and X-rays, and this effect became stronger as the thickness of the sample increased [12].

As is known [13], EPDM rubber is a material used in construction, roofing, hoses, adhesives and sealants. It is obtained from the polymerization process between ethylene, propylene and an unsaturated diene (5-ethylidene-2-norbornene) monomer. The saturated backbone structure provides chemical and climatic resistance. In addition, EPDM rubber is considered to be a cheap polymer, easy to process, with versatile applications. In the case of the graphene structure, it is known that it is a nanomaterial composed exclusively of *sp*^2^ hybridized carbon, in which the atoms are organized in a three-dimensional hexagonal network that adopts a honeycomb structure. The *π* bonds form a long-range conjugated network that confers a conductive character to graphene [14], obtaining rubbers with high electrical and thermal conductivity, and excellent mechanical properties [15,16]. Oils and polyethylene glycol (PEG) are other important additives used in rubbers to improve processability and achieve better compatibility between the filler and the rubber matrix [17,18]. In the case of PEG compounds, they have proven to be versatile and effective components capable of compatibilizing and increasing the interaction between filler and polymer. PEG can also act as an accelerator in a chemical process, allowing optimal and shorter crosslinking times to be achieved [19]. For example, it has been reported that the combined use of PEG and graphite in natural rubber compounds increases the tensile strength, electrical resistance and thermal stability of the rubber [20]. It has also been observed that PEG can act as a dispersant for silica fillers in a chloroprene rubber matrix, improving mechanical and vulcanization properties [21]. In addition, it has been reported that PEG promotes the intercalation of organomodified clay molecules in natural rubber, facilitating the dispersion of the filler and thus imparting better mechanical performance [22].

The aim of this research is to obtain a modified EPDM rubber incorporating graphene to increase the mechanical properties and bismuth (III) oxide to obtain a material capable of attenuating gamma radiation. The use of PEG to increase the interaction/dispersion of the filler and to improve the performance of the compound obtained is also discussed in this research.

## 2. Materials and Methods

### 2.1. Materials

The following materials were used to prepare the composite specimens. EPDM rubber KEP960N was obtained from Química Miralles S.A. Industry (Santiago, Chile). Zinc oxide (ZnO), stearic acid, powdered sulfur, N-cyclohexyl-2-benzothiazole sulfenamide (CBS), bismuth (III) oxide nanoparticles (size < 100 nm) and polyethylene glycol (PEG 1500, molar mass = 1500 Da) were purchased from Sigma Aldrich (St. Louis, MO, United States), and all reagents were ACS grade (≥95%). Graphene nanoplatelets (grain size > 50 μm) were imported from Xi’an Henrikang Biotech Co. Ltd. (Shanghai, China).

### 2.2. Composite Preparation

The EPDM rubber composites were prepared using a ZL-3018 two-roll mill, Zhongli Instrument Technology Co. Ltd. (Dongguan, China) according to the formulations described in Table 1 at room temperature for 20 min. The mixing consisted of dispersing the activators, filler and PEG into the EPDM rubber to facilitate the dispersion of the filler, followed by the addition of accelerator and sulfur. By considering that the attenuation properties to gamma radiation are determined by the atomic number of elements that make up the compound, bismuth (II) oxide was used as the filler with high content. In addition, graphene nanoplatelets were added in lower concentration, since they impart stiffness and inhibit the elasticity of the composites [23].

### 2.3. Characterization

The characteristics of the vulcanization systems, maximum torque (M_H_*)* and vulcanization time (t_90_), were determined using a ZL-3001 moving matrix rheometer (Zhongli Instrument Technology Co. Ltd., China) for 30 min at 180 °C. The vulcanization of the composites was performed using a ZL-3022 laboratory hydraulic press (Zhongli Instrument Technology Co. Ltd., China) for 18 min at 120 Kg/cm^2^ pressure at 180 °C [24]. The Abrasion Resistance Index (ARI) was measured using cylindrical dies of 1.6 × 1.6 × 0.8 cm according to ASTM Standard [25], and the hardness was determined using a rectangular die of 11.5 × 13 × 0.2 cm according to ASTM Standard [26].

The stress–strain test was determined using a Shimadzu EZ-X L 200 V instrument (Shimadzu, Kyoto, Japan) with a load cell of 500 N at 100 mm/min according to ASTM Standard [27].

The morphology of the samples was examined by scanning electron microscopy (SEM) using a Zeiss (Oberkocken, Germany) scanning electron microscope, model Gemini SEM 360, equipped with an Oxford Instrument (Abingdon, UK) EDS detector. The samples were covered with ultrathin gold (Au) film. The acceleration voltage was 10 kV.

The differential scanning calorimetry (DSC) of the different EPDM rubber compounds was recorded using a DSC 214 instrument (Netzsch, Selb, Germany) in the range between −100 °C and 100 °C with a heating rate of 10 °C/min. All samples were previously heated to 100 °C for two minutes to erase the thermal history. Thermogravimetric analysis (TGA) was performed in a temperature range of 25 to 800 °C using a Netzsch model Iris TG 209 F1 thermogravimetric analyzer in a nitrogen atmosphere at a heating rate of 10 °C/min.

A model FT/IR-4X spectrometer, Jasco (Tokyo, Japan), was used for FTIR-ATR analysis. Spectra were recorded in the range of 500 to 4000 cm^−1^.

For the measurement of gamma ray attenuation, disks with a diameter of 5 cm and a thickness varying between 0.1 mm and 10 mm were prepared. They were irradiated with a ^137^Cs source (661 keV), and the transmitted radiation was recorded with a hyperpure germanium detector based on the model CANBERRA GC 1018 Series B, corresponding to a multichannel Digital Spectrum Analyzer DSA (Canberra Industries, Meriden, CT, USA). The linear attenuation coefficient is determined by relating the transmitted radiation to the thickness of the samples using the following equation:

(1) $$N_{x}=N_{0}e^{-\mu x}$$

considering that the number of transmitted photons (*N*_*x*_) is equal to the number of incident photons (*N*_0_) raised to the attenuation coefficient (*μ*) times the thickness of the sample (*x*). In addition, the mass linear attenuation coefficient, which depends on the density of the material, is recorded in the same way, modifying Equation (1) as follows:

(2) $$\frac{\mu }{\delta }=\frac{1}{\delta x}\ln\frac{N_{0}}{N_{x}}$$

The HVL (Half Value Layer) is a parameter used to determine the thickness required for a material to reduce the intensity of radiation to half its initial intensity, given by Equation (3):

(3) $$\mathrm{HVL}=\frac{\ln2}{\mu }$$

Meanwhile, the thickness required for a material to reduce radiation to one-tenth of its original level is known as the Tenth Value Layer (TVL), expressed by Equation (4):

(4) $$\mathrm{TVL}=\frac{\ln10}{\mu }$$

Mean free path (MFP), transmission factor (TF) and radiation protection efficiency (RPE) were also calculated by using Equations (5)–(7).

(5) $$\mathrm{MFP}=\frac{1}{\mu }$$

(6) $$\mathrm{TF}=\frac{N_{x}}{N_{0}}$$

(7) $$\mathrm{RPE}\left(\%\right)=\left(1-\frac{N_{x}}{N_{o}}\right)\times 100$$

For Equations (6) and (7), the transmitted photons were registered for samples with a thickness of 10 mm.

## 3. Results and Discussion

The different formulations based on the EPDM polymer were obtained according to the procedures mentioned in the experimental section. When the term “unfilled EPDM” is mentioned in this paper, it is important to clarify that it deals with EPDM materials free of graphene nanoplatelets, bismuth oxide and PEG.

### 3.1. Curing Curves and Vulcanization Parameters

**Figure 1:**
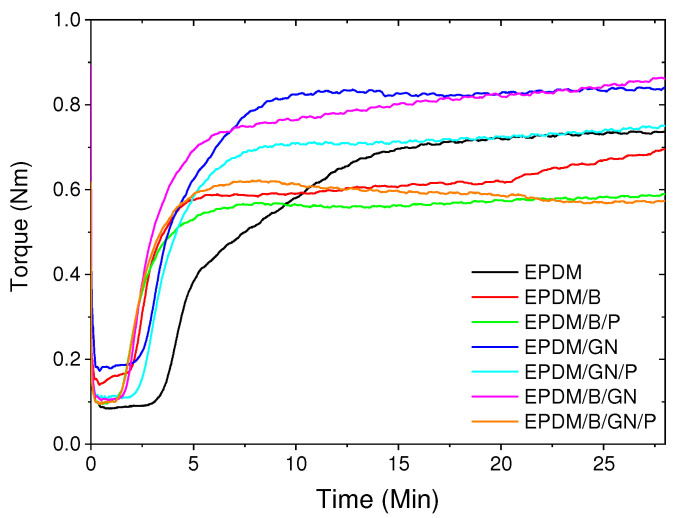
Curing curves of EPDM composites.

Figure 1 shows the rheometric curves of the different EPDM materials, and the values corresponding to the minimum torque (M_L_), maximum torque (M_H_), scorch time (t_s2_) and optimum curing time t_90_ are given in Table 2. According to the results, the incorporation of graphene nanoplatelets (GNs), bismuth (III) oxide and PEG to EPDM did slightly affect the different torques, depending on the nature of their interaction with the polymer matrix, as well as their morphological features. For instance, the effect of GNs on the M_H_ is explained by its laminar morphology, which makes a more significant contribution to increasing the viscosity of the sample [28,29]. This effect is not significantly affected by the present of PEG. In addition, bismuth (III) oxide nanoparticles did not induce changes in M_H_, which is attributed to their low volume fraction relative to the EPDM matrix.

On the other hand, the parameters t_s2_ and t_90_ decreased relatively when compared to those obtained for the unfilled EPDM. This effect is more pronounced in the presence of bismuth (III) oxide, except in the case of EPDM/B/GN material, which shows an increase in t_90_ of about 19% compared to the t_90_ of the unfilled EPDM. The effect on the t_90_ values is probably attributed to different contributions that either promote the decreasing effect or not. For instance, bismuth (III) oxide has interesting electronic properties; in fact, the optical basicity of bismuth (III) oxide could play a role in the vulcanization system based on CBS [30], where bismuth (III) oxide favors a more efficient crosslinking process, due to its ability to donate electrons to acidic species [31]. Graphene materials’ effects on the curing process have been reported and have been linked to electronic properties [32]. However, the evidence that t_90_ for the EPDM/B/GN sample was not lower than EPDM/B and comparable to that of EPDM/GN suggests the possibility that GNs and bismuth (III) oxide interfere with each other in the curing process. This hypothesis can be supported by the fact that EPDM/B/GN/P presented the lowest t_90_ value, indicating that PEG disrupts the interference between bismuth (III) oxide and GNs, and provides an additional effect on the crosslinking process due to its slight basicity, which is discussed below.

### 3.2. Mechanical Properties

**Figure 2:**
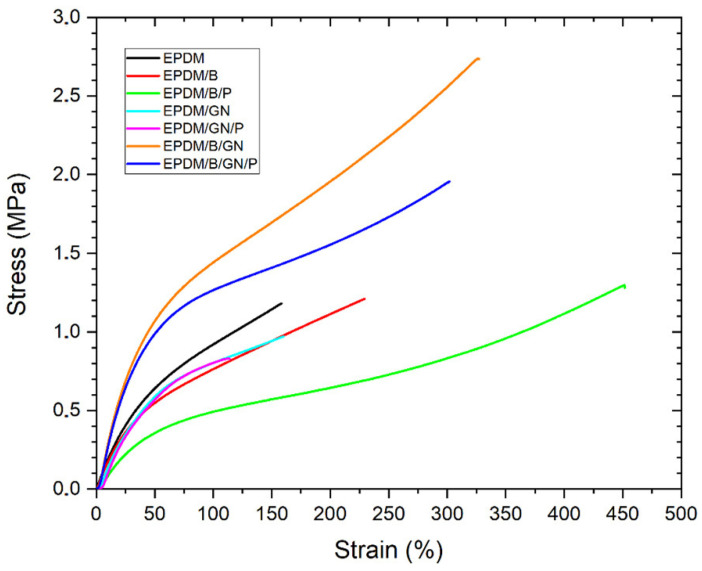
Stress–strain graphs (curves) of EPDM composites.

Tensile tests were performed on each EPDM-based composite, and the respective modulus values at 50% (E50) and 100% (E100) elongation, tensile stress and elongation at break are shown in Table 3, while the curves corresponding to the stress–strain analysis are shown in Figure 2.

The compound with the highest elongation is EPDM/B/P with 440%, followed by EPDM/B/GN/P and EPDM/B/GN compounds with 299% and 325%, respectively. The increase in elongation resulting from the addition of bismuth (III) as filler to NR/NBR rubber blend has been reported [33]. On the other hand, the EPDM/B/GN/P and EPDM/B/GN compounds have a higher maximum stress of 1.90 MPa and 2.65 MPa, respectively. The compounds with the lowest performance correspond to the composites containing graphene nanoplatelets, EPDM/GN and EPDM/GN/P with 157% and 114% elongation at break, respectively, giving values lower than those obtained for the unfilled EPDM.

All the composites with incorporated bismuth (III) oxide show a better mechanical performance than those without, indicating that bismuth (III) oxide would favor the crosslinking process. According to the literature [34], the strong interactions of p-d orbitals present in the bismuth atomic centers could contribute to the formation of species that promote the crosslinking of EPDM, using sulfur derivatives present in the unfilled material. This is also supported by the fact that the addition of bismuth (III) oxide to EPDM formulations reduced the optimum cure time (t_90_) of the EPDM-based mix compared to that of the unfilled EPDM (Table 2).

Table 4 shows the Abrasion Resistance Index (ARI) and hardness (Shore A) of the composites based on EPDM. Considering the ARI analysis, it is possible to observe an increase in this property for all the composites when compared to that obtained for the unfilled EPDM. The most significant increase is obtained for EPDM/B. Similarly, the increase in Shore A hardness is up to 18% for the EPDM/B/GN composite compared to that obtained for the unfilled EPDM.

The highest recorded values obtained for both Shore A hardness and ARI in compounds containing bismuth (III) oxide demonstrate a possible favorable affinity interaction, probably due to the hydrophobic nature of bismuth (III) oxide, which is also present in EPDM material [35,36]. Therefore, composites containing bismuth (III) oxide should exhibit greater adhesion to the polymer, imparting greater abrasion resistance.

### 3.3. FT-IR Spectroscopy Analysis

**Figure 3:**
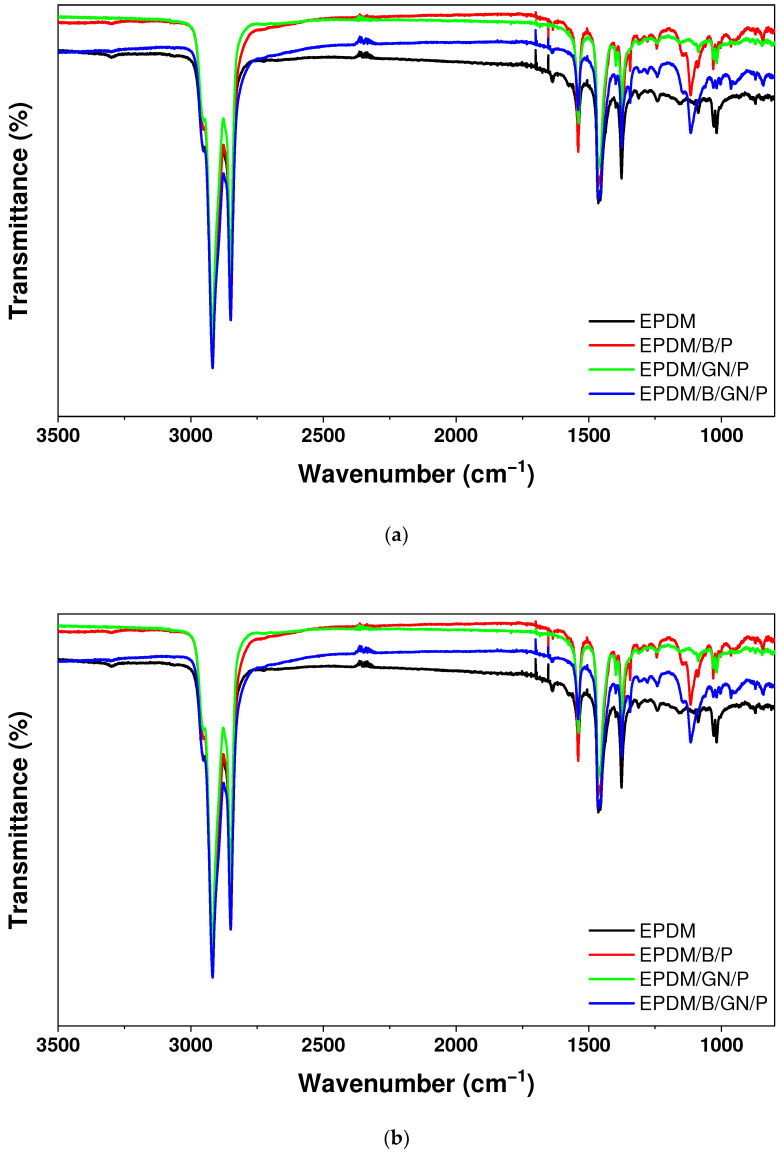

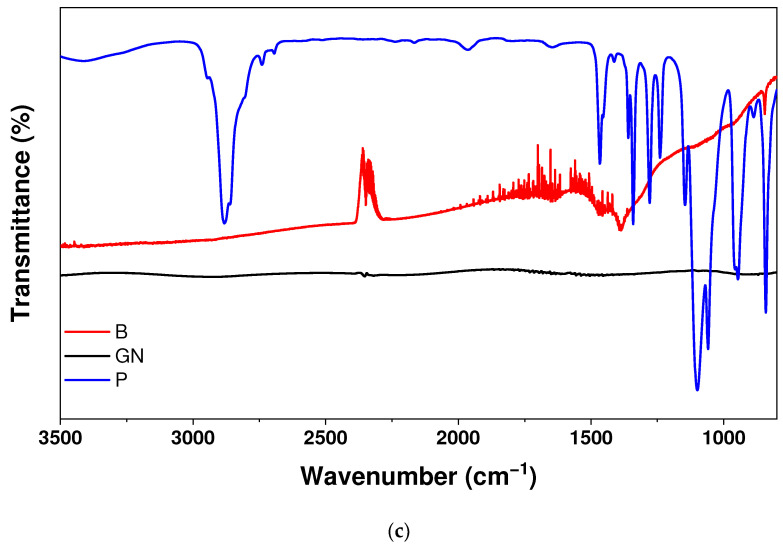
FT-IR spectra of (**a**) EPDM composites containing PEG as compatibilizer agent, (**b**) EPDM composites without PEG and (**c**) bismuth oxide, graphene nanoplatelets and PEG.

The FT-IR analysis was carried out, and the spectra of the unfilled EPDM and the different composites is shown in Figure 3. Concerning the unfilled EPDM, the signals observed at 2850 cm^−1^ and 2917 cm^−1^ correspond to the symmetrical and asymmetrical stretching of the C–H bonds present in the EPDM structure. In the range between around 700 cm^−1^ and 750 cm^−1^, it is possible to identify the absorption band corresponding to -(CH2-CH2)– backbone in the EPDM structure and the aromatic groups, confirming the presence of the CBS in the polymeric matrix, consistent with the report established by Silva, L., et al. [37] and Riba et al. [38]. In addition, the presence of N–H bonds belonging to the CBS structure is confirmed at 3300 cm^−1^. The incorporation of fillers such as bismuth oxide and graphene was confirmed by the FT-IR spectra. Indeed, for the composite EPDM/B, a stretching vibration observed at 680 cm^−1^ is attributed to the presence of Bi–O bonds [39,40].

An interesting result is deduced when comparing the spectra of the composites with and without PEG. As mentioned, the N-H stretching signal is present in the unfilled EPDM as seen in the EPDM/B, EPDM/GN and EPDM/B/GN composites. However, this signal at 3300 cm^−1^ disappears when PEG is incorporated in each respective composite. In addition, the O–H stretching band present at 3400 cm^−1^ in the PEG FT-IR spectrum [41] was not observed in the composites containing PEG. This result demonstrates that PEG not only contributes to the homogeneous dispersion of the filler in the EPDM matrix but also promotes the crosslinking process. Furthermore, the disappearance of the amine and hydroxyl signal present in the CBS and PEG compounds, respectively, [41,42] would indicate that there is an effective participation of this accelerator to form reactive polysulfides, and it could be interpreted as the slightly basic character [43] of PEG inducing a molecular interaction with the hydrogen atoms present in the nitrogenous species of CBS.

### 3.4. Thermal Properties

**Figure 4:**
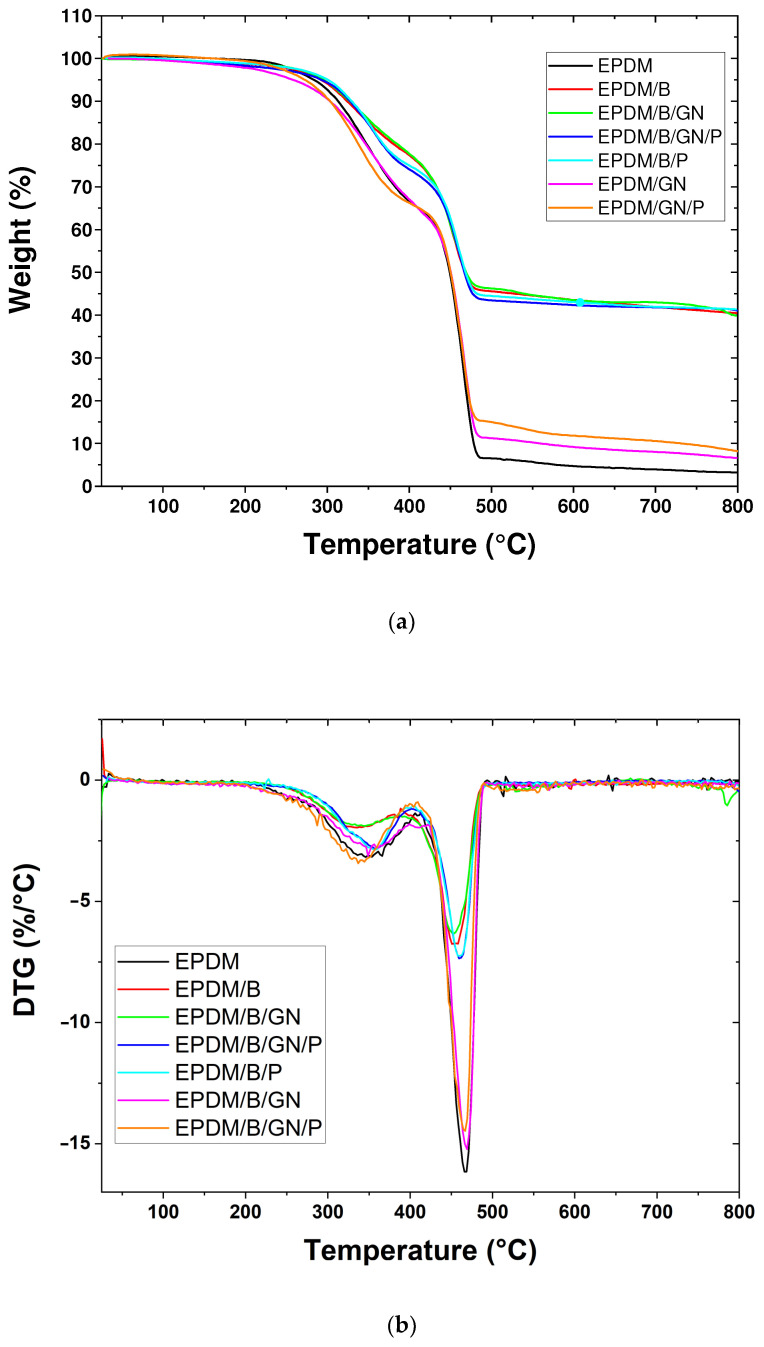
(**a**) Thermogravimetric analysis of EPDM composites and (**b**) the corresponding derivatives of the weight loss.

Figure 4 shows the TGA and DTG results of the analyzed EPDM rubber composites. Table 5 displays the values of the initial degradation temperature (Tid), maximum degradation temperature (Tmax), residual mass and glass transition temperature (Tg) of all composites. All products reach a Tmax over 400 °C. The compounds with bismuth present a residual mass close to 40%, suggesting that part of the oxygen present in the bismuth oxide was eliminated in the heating process. The graphene compounds have a residual mass of 6.55% and 7.74% corresponding to EPDM/GN and EPDM/GN/P, respectively. It is important to note that the glass transition temperature does not vary significantly with the content of the filler or the presence of the PEG additive [44,45].

Glass transition temperature (Tg) depicted in Table 5, obtained from DSC, did not change, a fact that indicates that PEG did not show a plasticizer effect [44], and according to the results presented above, it is more likely to have an effect on the crosslinking process. However, it is important to mention that the thermograms of the compounds containing PEG exhibited an endothermic process associated with the melting of PEG, which suggests segregation of this additive (see Supplementary Materials).

### 3.5. Morphology

**Figure 5:**
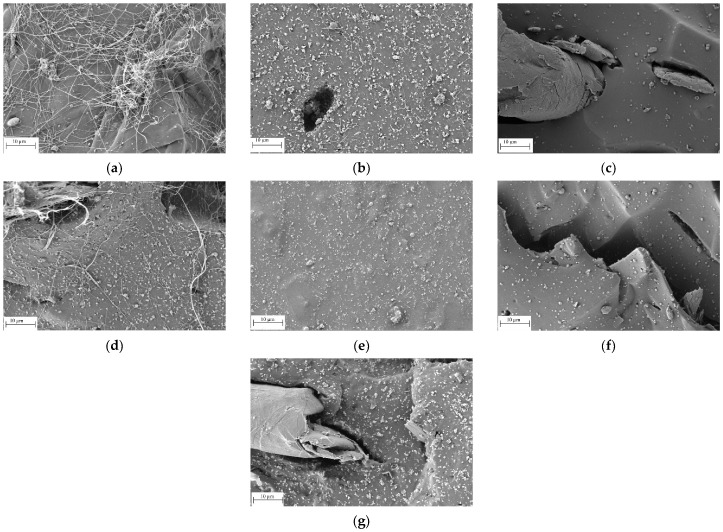
SEM images of the composites. (**a**) EPDM, (**b**) EPDM/B, (**c**) EPDM/GN, (**d**) EPDM/B/GN, (**e**) EPDM/B/P, (**f**) EPDM/GN/P and (**g**) EPDM/B/GN/P.

Figure 5 shows the SEM images obtained from the analysis of the cross section corresponding to the different EPDM composites subjected to mechanical tests. It is possible to observe that for the EPDM sample without filler content, shown in Figure 5a, there are filaments that indicate the plastic deformation experienced by the EDPM microdomains. It is interesting to note that the filler content significantly reduces the presence of these filaments, which are only slightly observed in the EPDM/B/GN composite. The main interpretation could be attributed to the fact that the presence of the fillers limits the mobility of the chains, preventing plastic deformation. In addition, it can be observed that the distribution of the fillers, especially the bismuth (III) oxide nanoparticles, is homogeneous. However, for the composites containing GNs, segregated domains are observed (Figure 5g).

**Figure 6:**
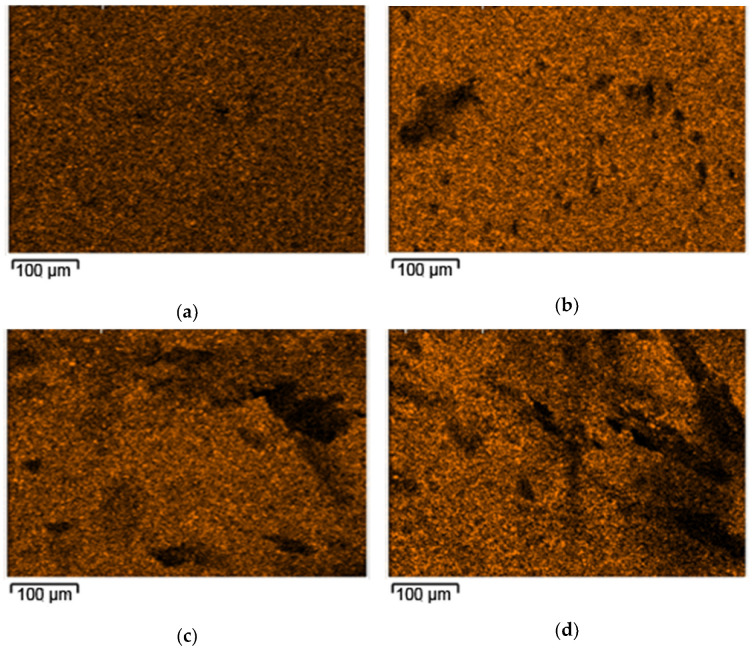
Energy-dispersive X-ray spectroscopy (EDS) mapping of Bi M α of EPDM/B (**a**), EPDM/B/P (**b**), EPDM/GN (**c**) and EPDM/GN/P (**d**).

In order to determine the distribution of the bismuth oxide dispersion in the composites, energy-dispersive X-ray spectroscopy (EDS) images were obtained for samples with and without GNs and/or PEG. The images showed that the dispersion of the bismuth oxide nanoparticles is affected by the presence of PEG or GNs. For example, Figure 6b presents zones with low content of bismuth oxide, but the more drastic evidence is for those composites containing a binary filler system. In fact, the fillers prevent each other from dispersing, resulting in a homogeneous dispersion. This indicates that the attenuation properties to gamma radiation can be affected by the presence of a binary system filler or other dispersants. The full analysis of the EDS mapping is available in the Supplementary Materials.

**Figure 7:**
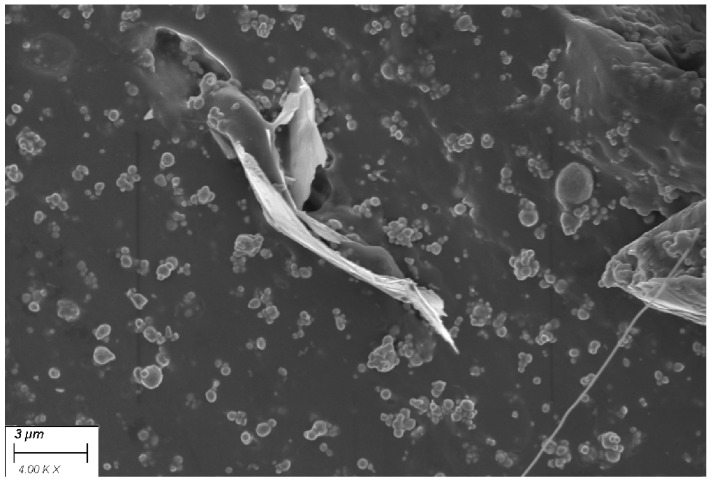
In-Lens mode SEM image of EPDM/B/GN/P.

Finally, in order to understand the distribution of both nanomaterials in the polymer matrix, EPDM/B/GN/P was analyzed using an immersion lens mode of (In-Lens). As it can be observed, Figure 7, which has a magnification of 4.00 k×, shows the zones of GN layers surrounded by spherical particles corresponding to bismuth (III) oxide. In fact, the continuous dispersion of these particles is interrupted by layers of graphene nanoplatelets.

### 3.6. High-Energy Electromagnetic Radiation Shielding Properties of EPDM-Based Composites

Table 6 displays the results of the linear attenuation coefficient µ, Tenth Value Layer (TVL), Half Value Layer (HVL), mass attenuation coefficient µ_mass_, the percentages of increase corresponding to the linear and mass attenuation coefficient versus the unfilled EPDM µ and µ_mass_, and finally the density of each EPDM composite. As reference, the linear attenuation coefficient of EPDM without filler is 0.0844 cm^−1^, consistent with that reported by other authors (0.082 cm^−1^) [46].

Composites containing bismuth (III) oxide increase their linear damping coefficient, with the EPDM/B compound showing the highest improvement, around 75%, compared to the unfilled EPDM. The presence of graphene nanoplatelets in the EPDM matrix, in the absence of bismuth oxide, did not really improve the linear attenuation coefficient since it is composed exclusively of carbon (Z = 6). In addition, EPDM/B/GN decreased the value of the linear damping coefficient, probably due to the formation of segregation zones of the bismuth (III) oxide nanoparticles, as shown in the SEM images of Figure 5, thus reducing or maintaining the µ-efficiency.

It is also observed that the composites, including graphene but without oxide bismuth (III), have a higher HVL value, explained by the density of the materials, which are for EPDM/GN and EPDM/GN/P 0.939 and 0.955 g/cm^3^, respectively, showing values lower than the unfilled EPDM. Essentially, it means that these materials need more mass to attenuate the same amount of radiation.

In order to understand the phenomena related to the changes in the attenuation properties, the pondered atomic number of the composites (${\bar{Z}}_{c}$) was determined (Equation (8)) [23].

(8) $${\bar{Z}}_{c}=\sum \left(Z_{i}\frac{N_{i}}{N}\right)$$

where *Z*_*i*_ and *N*_*i*_ are the atomic number and number of atoms of each element, and *N* corresponds to the total number of atoms.

For example, EDPM/B/GN has the highest value of ${\bar{Z}}_{c}$ (4.89), but its mass attenuation coefficient decreased by 5%, while EPDM/B/GN/P has a ${\bar{Z}}_{c}$ of 4.80, and the increase in mass attenuation coefficient is of 9% with respect to EPDM. This fact suggests that the dispersion of high-atomic-number nanoparticles such as bismuth (III) oxide influences the attenuation properties. It is interesting to note that polyethylene glycol (P) has a positive effect on those composites containing GNs, and in contrast, for the composite containing only bismuth oxide as filler, P discretely inhibits its attenuation properties. This is also corroborated by other parameters depicted in Table 6, such as MPF and PRE.

It is well known that gamma radiation interacts with matter through absorption or scattering phenomena, and for elastomer-based composites, as demonstrated in this research, the dispersion of high-atomic-number fillers is essential to achieve higher shielding properties. The homogeneous filler dispersion increases the occurrence probability of these phenomena [23].

## 4. Conclusions

In this study, very promising results were obtained regarding the effect of bismuth (III) oxide, polyethylene glycol and graphene nanoplatelets on the mechanical, vulcanization and gamma radiation attenuation properties of EPDM rubber. It has been shown that the presence of PEG 1500 improves the dispersion of the filler. Furthermore, a reduction of about 18% in the t_s2_ and t_90_ times was observed for the composites containing bismuth (III) oxide when compared to those obtained for the unfilled EPDM. Finally, it was possible to demonstrate that EPDM composites in the presence of bismuth (III) oxide are able to behave as a shield against high-energy electromagnetic radiation. The EPDM/B composite shows an improvement up to 75%, this composite being the one with the best performance among all the composites studied in this investigation, followed by EPDM/B/GN/P with a 61% improvement if compared to unfilled EPDM. However, the presence of graphene promotes an irregular distribution of the filler content in the matrix, leading to the formation of segregation zones, as visualized in the SEM images. The next challenge of this research will be to find chemical solutions to improve the filler distribution in this type of composite, with the aim of obtaining better physicochemical properties.

## Figures and Tables

**Table 1:** EPDM composite formulations.

Compound	Function	Unfilled EPDM	Composite EPDM/B/P	Composite EPDM/GN/P	Composite EPDM/B	Composite EPDM/GN	Composite EPDM/B/GN/P	Composite EPDM/B/GN
EPDM	Polymer matrix	100	100	100	100	100	100	100
ZnO	Activator	5	5	5	5	5	5	5
Stearic acid	Activator	2	2	2	2	2	2	2
CBS	Accelerator	7.8	7.8	7.8	7.8	7.8	7.8	7.8
Graphene	Filler	-	-	10	-	10	10	10
Bi_2_O_3_	Filler	-	100	-	100	-	100	100
PEG1500	Dispersing agent	-	10	1	-	-	11	-
Sulfur powder	Crosslinking agent	1.7	1.7	1.7	1.7	1.7	1.7	1.7

**Table 2:** Curing data of EPDM composites.

Material	M_L_ (Nm)	M_H_ (Nm)	t_s2_ (min)	t_90_ (min)
EPDM	0.850 ± 0.001	0.735 ± 0.093	4.76 ± 0.70	11.46 ± 1.62
EPDM/B/P	0.950 ± 0.002	0.537 ± 0.090	1.92 ± 0.23	5.22 ± 2.63
EPDM/B	0.135 ± 0.099	0.625 ± 0.053	2.63 ± 0.01	4.06 ± 0.33
EPDM/GN/P	0.135 ± 0.029	0.824 ± 0.059	2.08 ± 0.31	6.30 ± 1.48
EPDM/GN	0.164 ± 0.027	0.819 ± 0.023	3.43 ± 0.45	7.58 ± 0.75
EPDM/B/GN/P	0.930 ± 0.003	0.618 ± 0.064	1.86 ± 0.28	3.58 ± 0.87
EPDM/B/GN	0.104 ± 0.001	0.750 ± 0.053	2.01 ± 0.20	6.83 ± 0.98

**Table 3:** Modulus at 50% of elongation (E50), modulus at 100% of elongation (E100), tensile strength and elongation at break of EPDM composites.

Material	E 50 (MPa)	E 100 (MPa)	Tensile Strength (MPa)	Elongation at Break (%)
EPDM	0.63 ± 0.02	0.90 ± 0.03	1.19 ± 0.11	173 ± 16
EPDM/B/P	0.35 ± 0.01	0.50 ± 0.01	1.28 ± 0.13	440 ± 39
EPDM/B	0.58 ± 0.08	0.81 ± 0.12	1.27 ± 0.09	235 ± 25
EPDM/GN/P	1.27 ± 0.03	0.81 ± 0.01	0.84 ± 0.01	114 ± 1
EPDM/GN	0.54 ± 0.06	0.76 ± 0.06	0.91 ± 0.07	157 ± 6
EPDM/B/GN/P	0.95 ± 0.04	1.23 ± 0.04	1.90 ± 0.24	299 ± 40
EPDM/B/GN	1.03 ± 0.04	1.41 ± 0.04	2.63 ± 0.14	325 ± 24

**Table 4:** Abrasion resistance (ARI) and hardness (Shore A) of the EPDM composites.

Material	Hardness (Shore A)	ARI (%)
EPDM	39.8 ± 0.76	39.08
EPDM/B/P	38.8 ± 0.29	67.21
EPDM/B	44.0 ± 0.58	88.37
EPDM/GN/P	44.5 ± 0.29	59.29
EPDM/GN	44.5 ± 0.29	44.02
EPDM/B/GN/P	44.3 ± 0.76	47.46
EPDM/B/GN	47.3 ± 0.29	75.46

**Table 5:** TGA data and glass transition temperature of EPDM composites.

Composites	Tid (°C)	Tmax (°C)	Residual Mass at 800 °C (%)	Tg (°C)
EPDM	345.4	467.1	3.18	−52.45
EPDM/B	335.3	452.1	40.45	−53.06
EPDM/GN	348.5	468.0	6.55	−52.45
EPDM/B/P	356.1	458.8	41.26	−52.45
EPDM/GN/P	337.0	466.1	7.74	−52.07
EPDM/B/GN	344.0	452.6	39.78	−52.08
EPDM/B/GN/P	356.8	459.7	41.02	−52.45

**Table 6:** Density (*ρ*), linear attenuation coefficient (µ), percentage of µ increase, mean free path (MFP), Tenth Value Layer (TVL), Half Value Layer (HVL), transmission factor (TF), radiation protection efficiency (RPE), mass attenuation coefficient µ_mass_ and percentages of increase in mass attenuation coefficient of each EPDM composites.

Sample	*ρ* (g/cm^3^)	µ (cm^−1^)	µ _Increase_ (%)	MFP (cm)	TVL (cm)	HVL (cm)	TF	RPE (%)	µ_mass_ _(g/cm_^2^_)_	µ_mass Increase_ (%)	${\bar{Z}}_{c}$
EPDM	1.014	0.0844		11.8	8.21	27.3	0.907	9.25	0.0832		3.11
EPDM/B	1.516	0.147	75	6.80	4.71	15.6	0.846	15.4	0.0972	17	4.85
EPDM/GN	0.939	0.0612	−27	16.3	11.33	37.6	0.933	6.75	0.0652	−22	3.22
EPDM/B/GN	1.664	0.131	55	7.63	5.29	17.6	0.866	13.4	0.0787	−5	4.89
EPDM/B/P	1.418	0.129	53	7.75	5.37	17.8	0.871	12.9	0.0910	9	4.77
EPDM/GN/P	0.955	0.0837	−1	11.9	8.28	27.5	0.911	8.92	0.0876	5	3.22
EPDM/B/GN/P	1.500	0.136	61	7.35	5.10	16.9	0.867	13.3	0.0907	9	4.80

## Data Availability

Data are contained within the article and Supplementary Materials. The data that support the findings of this study are available on request from the corresponding author, H.A.-B.

## Supplementary Materials

The following supporting information can be downloaded at: https://www.mdpi.com/article/10.3390/polym16050633/s1, Figure S1: Differential scanning calorimetry of EPDM-based composites in the range between −100 °C and 100 °C; Figure S2: Energy-dispersive X-ray spectroscopy (EDS) mapping of EPDM/Bi; Figure S3: Energy-dispersive X-ray spectroscopy (EDS) mapping of EPDM/Bi/P; Figure S4: Energy-dispersive X-ray spectroscopy (EDS) mapping of EPDM/Bi/GN; Figure S5: Energy-dispersive X-ray spectroscopy (EDS) mapping of EPDM/Bi/GN/P.
