# Supplementary material for: Design and Study of Novel Composites Based on EPDM Rubber Containing Bismuth (III) Oxide and Graphene Nanoplatelets for Gamma Radiation Shielding
Source: Polymers (Basel). 2024 Feb 26;16(5):633. doi: 10.3390/polym16050633 (PMC10935033; doi:10.3390/polym16050633)
Supplement: Supplementary file 1 [file polymers-16-00633-s001.zip › polymers-2868524-supplementary.pdf]

Supplementary Materials

Design and Study of Novel Composites Based on EPDM Rubber Containing Bismuth (III) Oxide and Graphene Nanoplatelets for Gamma Radiation Shielding

Gabriela Álvarez-Cortez [1], Francisco Molina [2,3], Bruno F. Urbano [1], Mohamed Dahrouch [4], Marianella Hernández Santana [5], Miguel A. Lopez Manchado [5], Raquel Verdejo [5] and Héctor Aguilar Bolados [1,*]

[1] Departamento de Polímeros, Facultad de Ciencias Químicas, Universidad de Concepción,
Concepcion 3349001, Chile; galvarez@udec.cl (G.Á.-C.); burbano@udec.cl (B.F.U.)
[2] Centro de Investigación en Física Nuclear y Espectroscopia de Neutrones CEFNen, Comisión Chilena de Energía Nuclear, Santiago, Chile; francisco.molina@cchen.cl
[3] Millennium Institute for Subatomic Physics at High Energy Frontier—SAPHIR, Santiago, Chile
[4] Departamento de Química Orgánica, Facultad de Ciencias Químicas, Universidad de Concepción,
Concepcion 3349001, Chile; mdahrouch@udec.cl
[5] Instituto de Ciencia y Tecnología de Polímeros (ICTP), CSIC, Juan de la Cierva, 3, 28006-Madrid, Spain; marherna@ictp.csic.es (M.H.S.); lmanchado@ictp.csic.es (M.A.L.M.); r.verdejo@csic.es (R.V.)
* Correspondence: haguilar@udec.cl

Differential Scanning Calorimetry

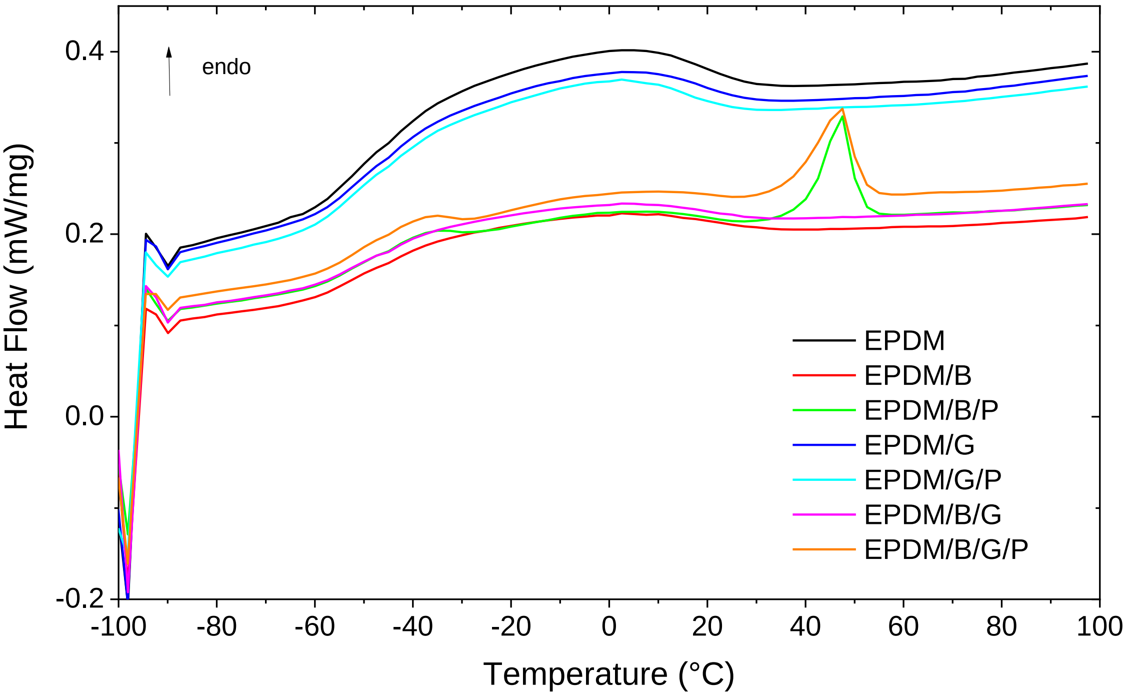

Figure S1. Differential scanning calorimetry of EPDM-based composites in the range between -100°C and 100°C.

Energy dispersive X-ray spectroscopy (EDS) mapping

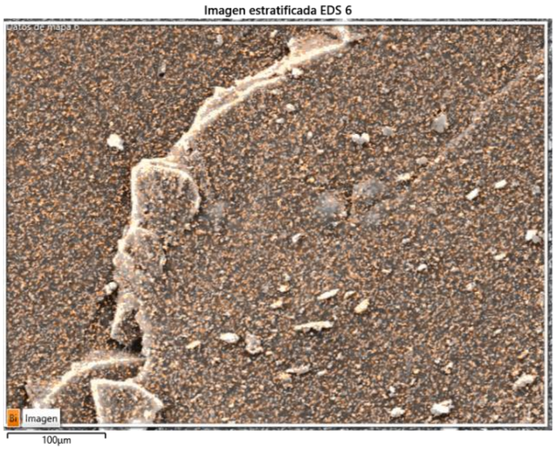

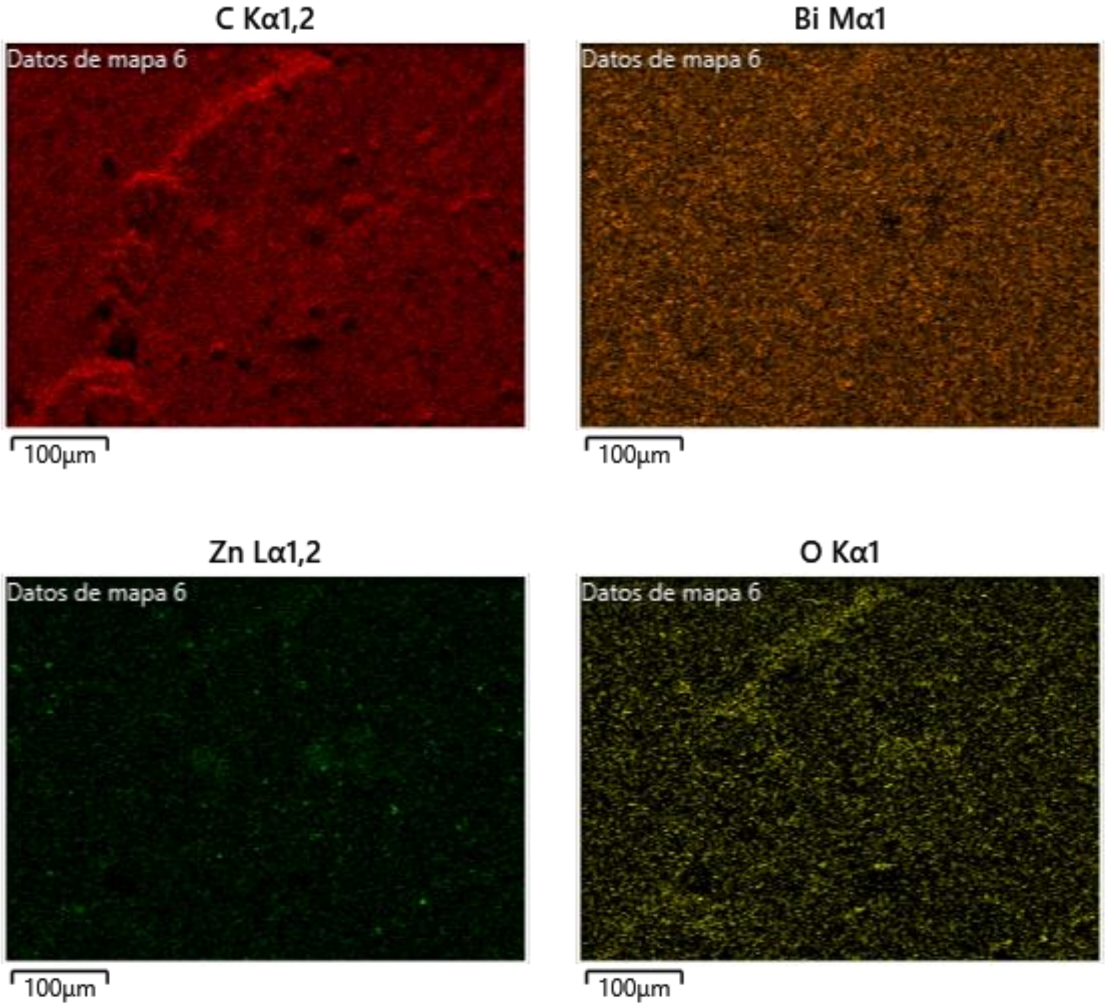

Figure S2. Energy dispersive X-ray spectroscopy (EDS) mapping of of EPDM/Bi

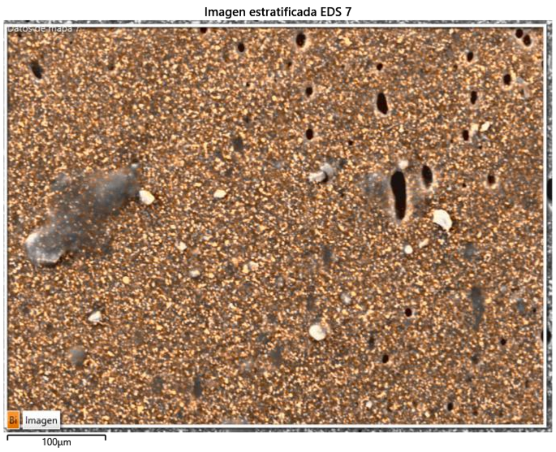

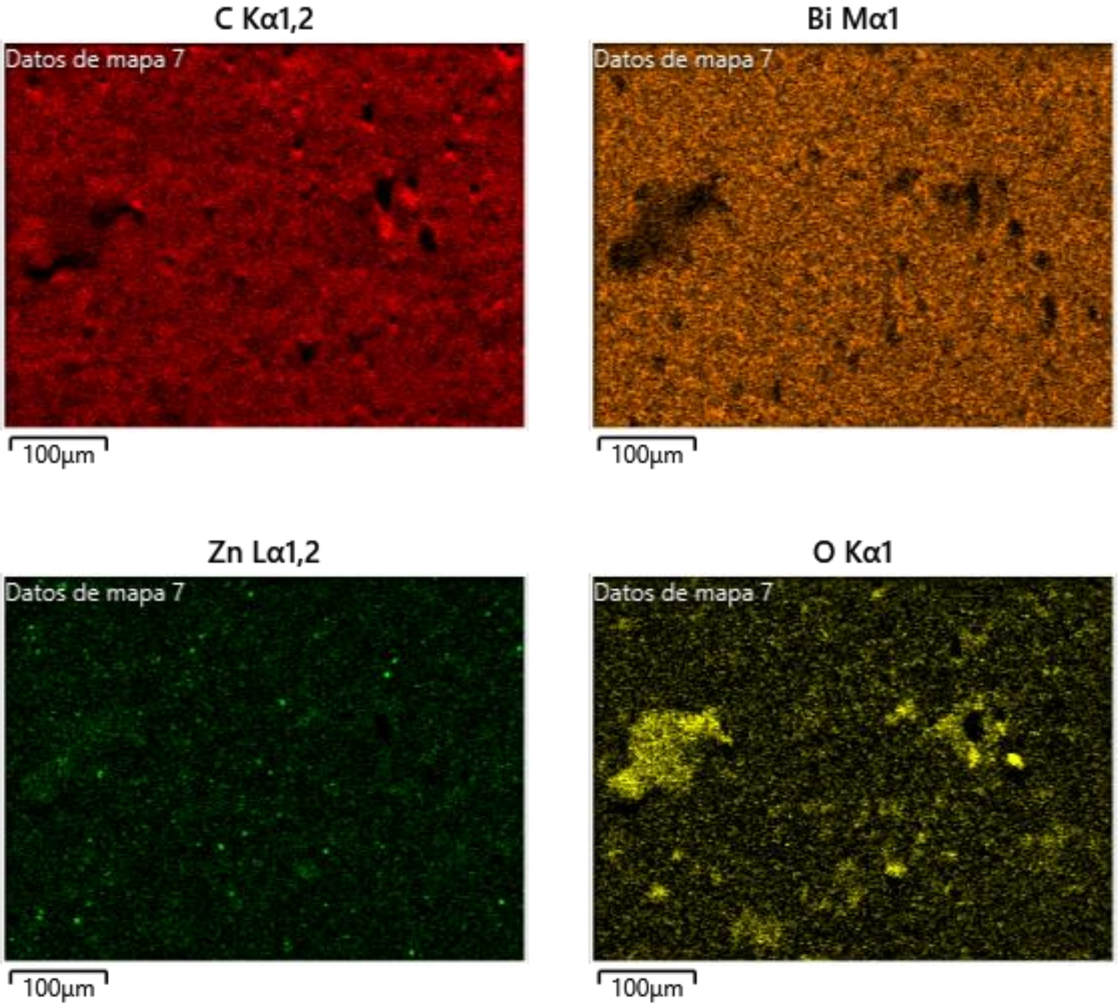

Figure S3. Energy dispersive X-ray spectroscopy (EDS) mapping of of EPDM/Bi/P

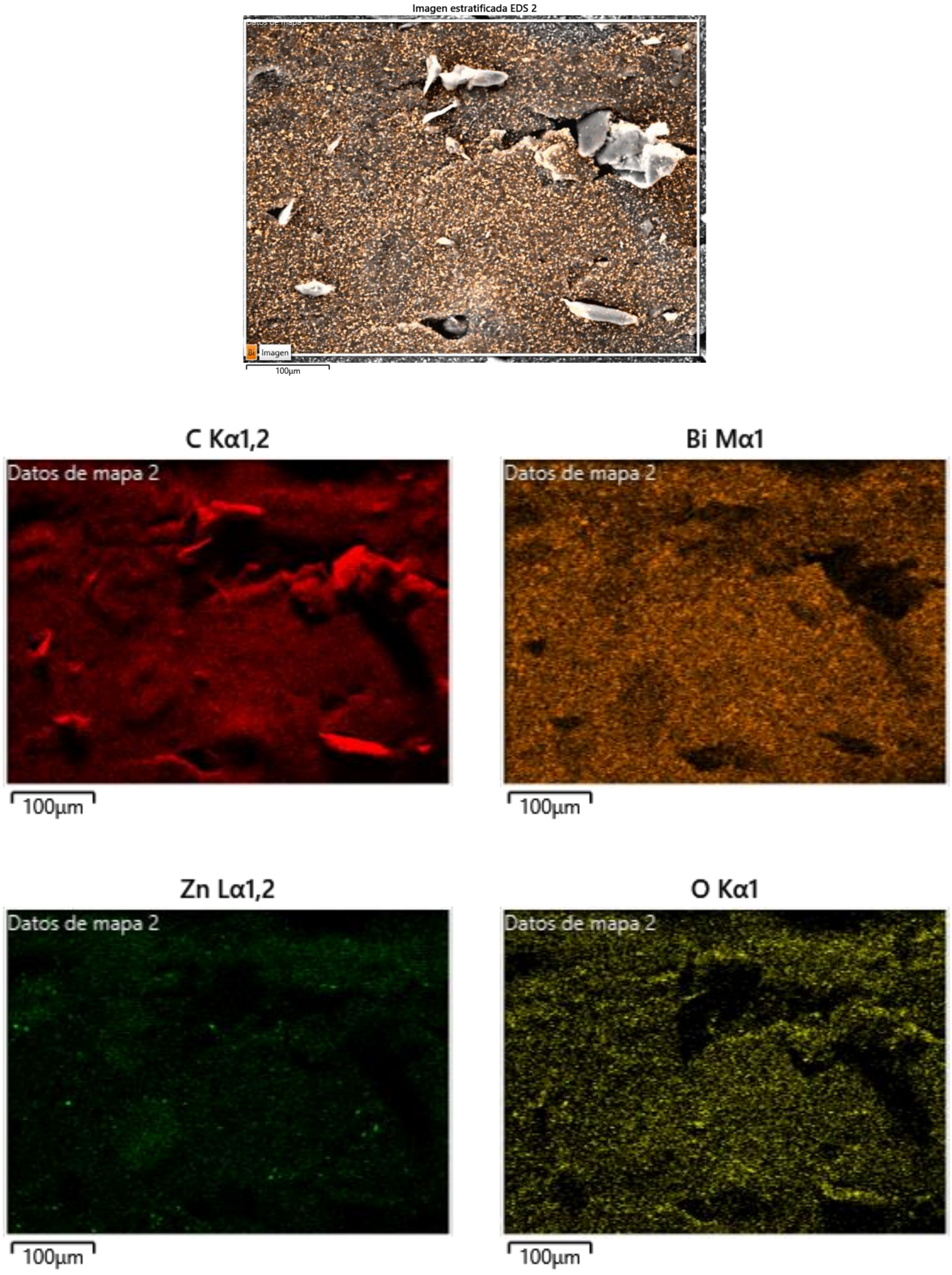

Figure S4. Energy dispersive X-ray spectroscopy (EDS) mapping of of EPDM/Bi/GN

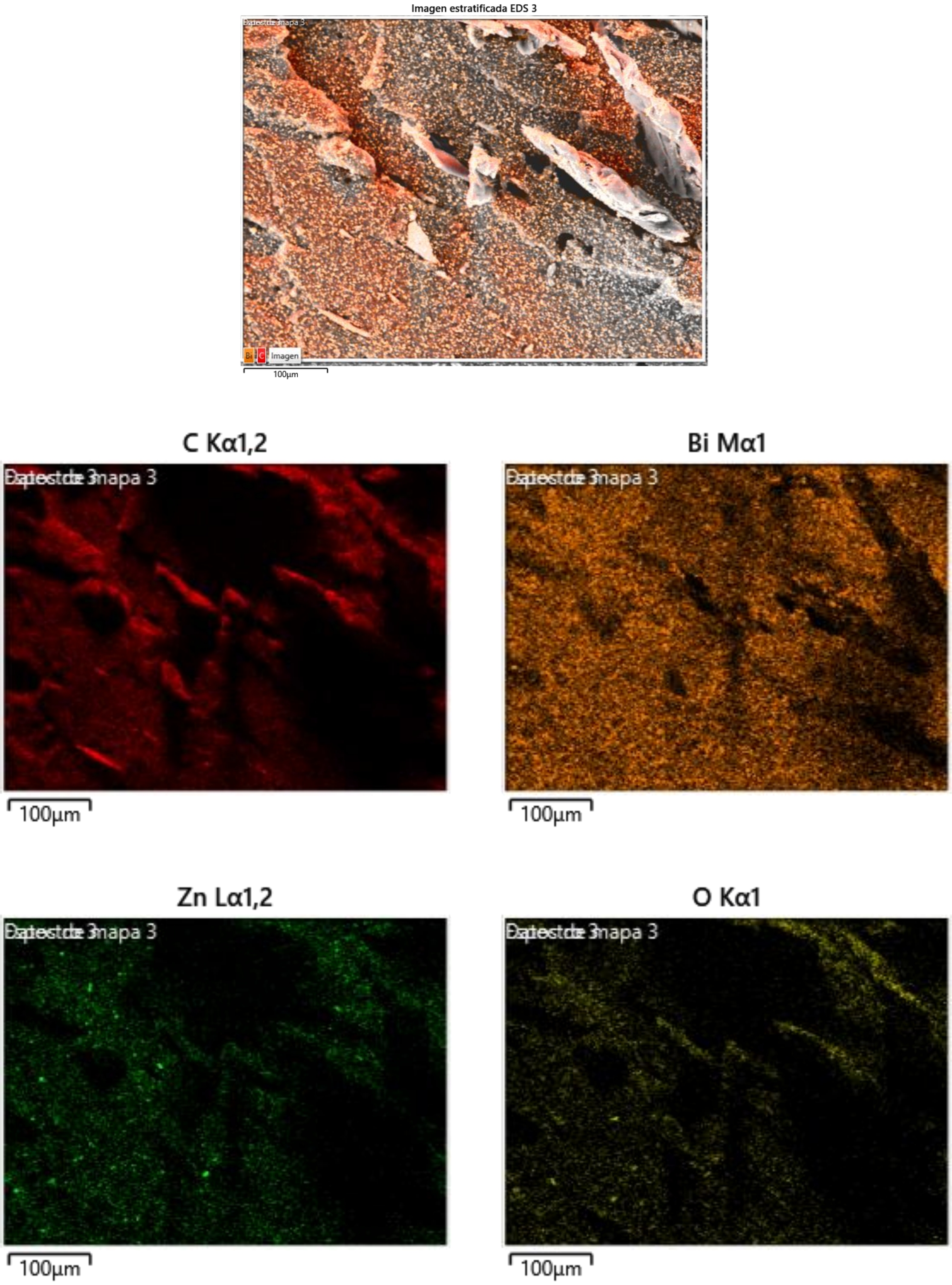

Figure S5. Energy dispersive X-ray spectroscopy (EDS) mapping of of EPDM/Bi/GN/P
